# Current accuracy of surface matching compared to adhesive markers in patient-to-image registration

**DOI:** 10.1007/s00701-019-03867-8

**Published:** 2019-03-16

**Authors:** Mireli A. Mongen, Peter W. A. Willems

**Affiliations:** 1 Department of Neurosurgery, Leiden University Medical Center, Leiden, The Netherlands; 2 Departmesnt of Neurosurgery, University Medical Center Utrecht, Internal Postage G03.124, PO-box 85500, 3584 CX Utrecht, The Netherlands

**Keywords:** Frameless stereotaxy, Rigid body transformation, Application accuracy, Surface matching, Point-pair matching

## Abstract

**Object:**

In the past, the accuracy of surface matching has been shown to be disappointing. We aimed to determine whether this had improved over the years by assessing application accuracy of current navigation systems, using either surface matching or point-pair matching.

**Methods:**

Eleven patients, scheduled for intracranial surgery, were included in this study after a power analysis had shown this small number to be sufficient. Prior to surgery, one additional fiducial marker was placed on the scalp, the “target marker,” where the entry point of surgery was to be expected. Using one of three different navigation systems, two patient-to-image registration procedures were performed: one based on surface matching and one based on point-pair matching. Each registration procedure was followed by the digitization of the target marker’s location, allowing calculation of the target registration error. If the system offered surface matching improvement, this was always used; and for the two systems that routinely offer an estimate of neuronavigation accuracy, this was also recorded.

**Results:**

The error in localizing the target marker using point-pair matching or surface matching was respectively 2.49 mm and 5.35 mm, on average (*p* < 0.001). In those four cases where an attempt was made to improve the surface matching, the error increased to 6.35 mm, on average. For the seven cases where the system estimated accuracy, this estimate did not correlate with target registration error (*R*^2^ = 0.04, *p* = 0.67).

**Conclusion:**

The accuracy of navigation systems has not improved over the last decade, with surface matching consistently yielding errors that are twice as large as when point-pair matching with adhesive markers is used. These errors are not reliably reflected by the systems own prediction, when offered. These results are important to make an informed choice between image-to-patient registration strategies, depending on the type of surgery at hand.

## Introduction

Since its introduction in the 1980s, frameless stereotaxy, or neuronavigation, has become increasingly popular and has now become an integral part of intracranial surgery. In principle, neuronavigation determines and demonstrates the relationship between preoperative imaging data, typically computed tomographic (CT) and/or magnetic resonance (MR) images, and the intraoperative position of a surgical instrument. The neuronavigation computer finds this relationship with a patient-to-image registration procedure based on a rigid body transformation. A rigid body transformation finds the best “fit” between two coordinate systems, in this case, image coordinates and operating room (OR) coordinates, without distorting either coordinate system. Typically, this procedure uses either point-pair matching or surface matching to find this “fit.”

Although anatomical landmarks may be used to perform point-pair matching, they are relatively difficult to digitize accurately, not abundantly available on a human head, and those that are available are not widely distributed (ears, eyes, and nose). To overcome these limitations, adhesive markers can be applied to the scalp, prior to image acquisition, which remain in place until they are digitized in OR space. As a consequence, their use requires additional preoperative image acquisition. To circumvent the costs of adhesive markers and the necessity for additional imaging, without reverting to anatomical landmarks, one may also use surface matching. In surface matching, a cloud of randomly digitized points on the scalp is fitted to a volume rendering of the imaging data.

The accuracy with which the image and OR coordinate systems are coregistered impacts directly on the application accuracy during surgery. Unfortunately, in previous comparisons of patient-to-image registration techniques, surface matching was found to be significantly less accurate than point-pair matching with either anatomical landmarks or adhesive markers [3, 13, 14]. Despite these reports, surface matching is still available on modern neuronavigation systems and appears to gain popularity. Partly, this may be due to its advantages mentioned above, i.e., ease of use when compared to anatomical landmarks and cost-effectiveness when compared to adhesive markers, but unawareness about the inaccuracy of surface matching may also contribute. Furthermore, a belief may settle in, that software advances have led to improved surface matching algorithms, which no longer suffer the inaccuracies reported earlier.

We aimed to determine the current application accuracy of neuronavigation, using patient-to-image registration based on either surface matching or point-pair matching with adhesive markers, using three different commercially available navigation systems.

## Materials and methods

### Patient inclusion

A power calculation was performed, based on a paired *T* test analysis, with a type I error rate of 0.05 and a type II error rate of 0.1, using the results of our earlier publication [14]: an effect difference of 2.5 with a standard deviation of 2.0. This resulted in seven patients, scheduled for intracranial surgery, to be included. These were included in one center (Leiden UMC) and operated using either the StealthStation S7 (four cases) or the newer StealthStation S8 (three cases) neuronavigation system (both from Medtronic, Minneapolis, USA). To assess whether the results were specific to one vendor, additionally, four patients were included at another center (UMC Utrecht) and operated using the Curve neuronavigation system (Brainlab AG, Munich, Germany).

### Patient-to-image registration procedures

**Fig. 1:**
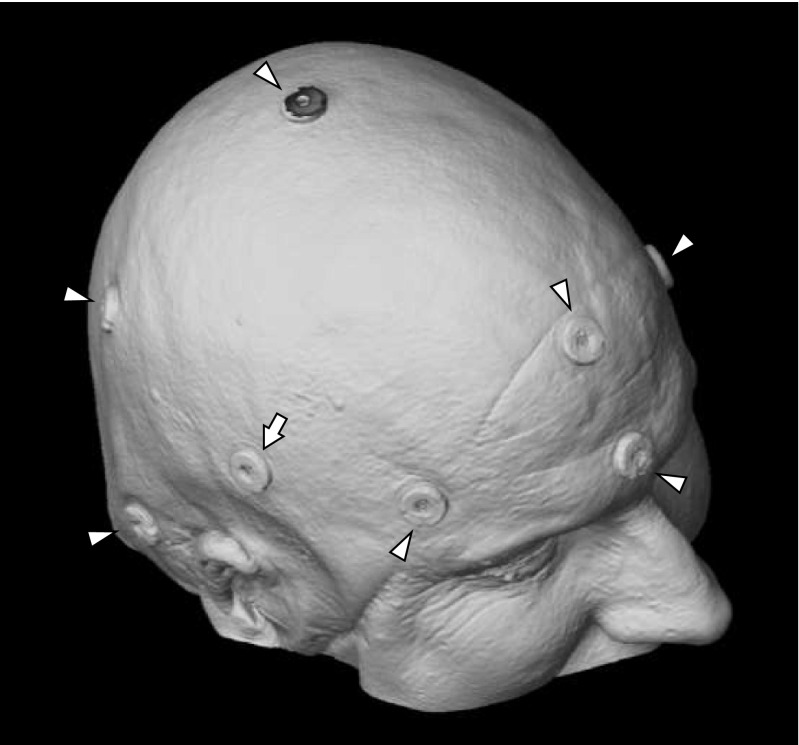
Three-dimensional MRI reconstruction demonstrating a typical distribution of adhesive markers. The seven markers for registration are indicated by arrowheads. The arrow depicts the additional “target-marker” where surgical entry is to be expected (close to the pathology). In this case, the Curve was used for navigation, and TRE values were as follows: adhesive markers, 3.30 mm; surface matching, 6.00 mm; and second surface matching attempt, 6.30 mm

In accordance with our routine clinical protocols, between 5 and 7 adhesive markers were applied to the scalp, 1 day preoperatively. In addition, a supplementary marker, the “target-marker,” was applied where the surgical entry was to be expected, i.e., as close as possible to the pathology (Fig. 1). MR imaging was then performed, and the images were transferred to the neuronavigation system. All markers were digitized manually, in image space.

In the operating room, the patient’s head was immobilized with a Mayfield head clamp, and two separate patient-to-image registration procedures were performed. First, surface matching was performed, according to each system’s specific requirements. The target-marker was then digitized in OR space with a regular pointer device and its corresponding image coordinates recorded. Second, point-pair matching was performed with all adhesive markers except the target-marker. Again, the target marker was digitized in OR space and its corresponding image coordinates recorded. The StealthStation systems (S7 and S8) both offered an estimation of registration accuracy (ERA) after point-pair matching, which was also recorded.

The target registration error (TRE), the error with which the navigation system identifies the location of the target-marker, was calculated as the Euclidean distance between the position of the marker as manually digitized in image space and the position of the marker as digitized with the pointer device. The StealthStation systems offered *X*-, *Y*- and *Z*-coordinates of both positions, allowing calculation of the distance as follows: ((*X*_image_ − *X*_pointer_)^2^ + (*Y*_image_ − *Y*_pointer_)^2^ + (*Z*_image_ − *Z*_pointer_)^2^)^1/2^. The Curve offered the distance automatically.

Finally, the Curve offered the option to improve surface matching by adding a second point cloud. After recording the initial TRE with surface matching, this option was always used, and the resulting new TRE was also recorded.

### Statistical analysis

TRE values between registration strategies were compared using paired *T* tests. For point-pair registration with the StealthStation systems (S7 and S8), correlation between the ERA and TRE values was calculated using linear regression analysis.

## Results

Eleven patients were included as detailed above, between November 2017 and July 2018. Most of these had either a frontal (5 patients) or temporal (3 patients) location for the target marker. There were two occipital locations and one parietal location.

**Figure 2:**
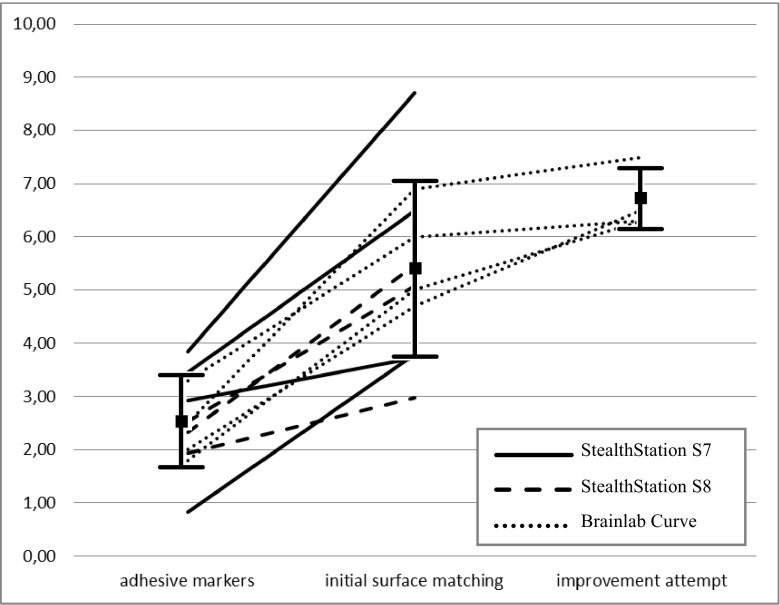
Graph showing all TRE measurements (in mm) using adhesive markers, surface matching, and a secondary surface matching attempt. The latter was only available in the Curve navigation system. Each line represents different measurements within a single patient. Line styles are explained in the legend. I-bars represent means ± standard deviation

**Figure 3:**
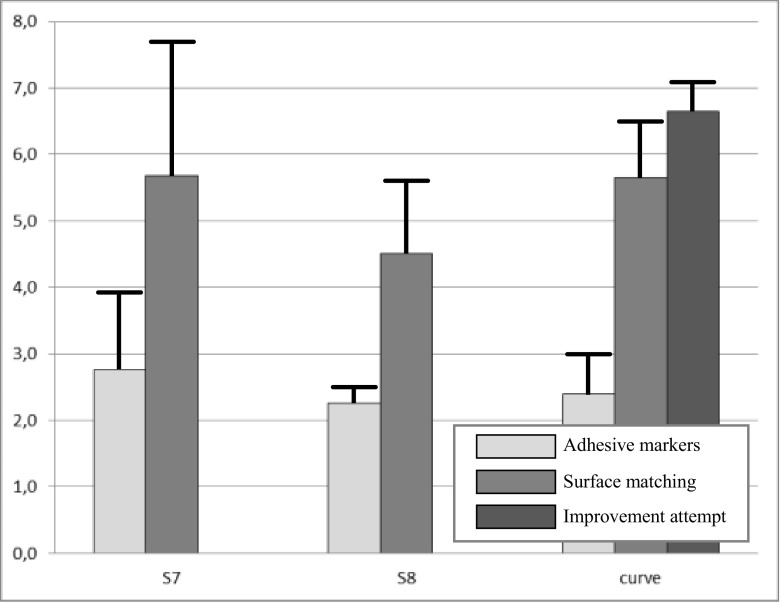
Graph showing the mean TRE values (T-bar: mean + SD) using adhesive markers or surface matching per navigation system. Again, a second surface matching attempt (improvement attempt) is shown for the Curve navigation system

TRE measurements are offered in Fig. 2. In all cases, surface matching was significantly less accurate than point-based registration (*p* < 0.001). On average (±SD), TRE after point-based registration was 2.49 ± 0.86 mm and TRE after surface matching was 5.35 ± 1.64 mm. The mean difference in accuracy was 2.85 (± 1.20) mm. There was no obvious difference between navigation systems, regarding these results (Fig. 3). Moreover, in all cases with the Curve, the second surface matching attempt worsened accuracy, rather than improved it. This further degradation was, on average, 1.00 ± 0.68 mm (*p* = 0.06).

**Figure 4:**
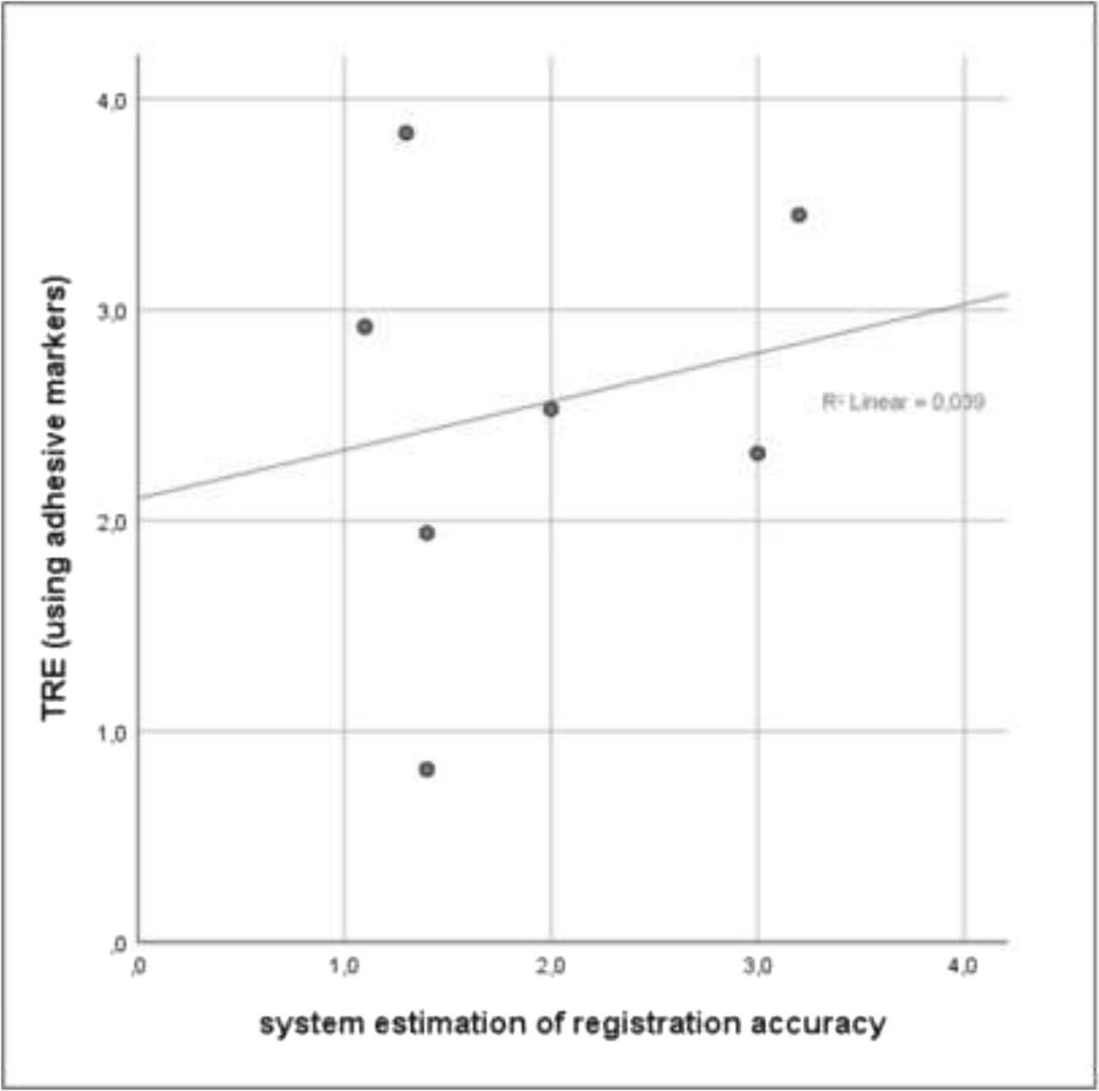
Scatterplot showing the relationship between the system’s estimation of registration accuracy (ERA) and the TRE. This concerns the estimations by the StealthStation systems (S7 or S8) after point-based registration, using adhesive markers. Poor correlation between estimation and TRE is clearly demonstrated

For the StealthStation cases, Fig. 4 demonstrates lack of correlation between ERA values and TRE values (*R*^2^ = 0.04, *p* = 0.67).

## Discussion

In this paper, we present a clinical evaluation of the application accuracy of current neuronavigation systems, comparing the use of adhesive markers with that of surface matching. Our results clearly demonstrate that the development of neuronavigation systems over the last decade has not led to increased accuracy of either technique. More importantly, surface matching is still significantly inferior to point-pair matching with regard to accuracy, regardless of which navigation system is used. Furthermore, we demonstrate that the estimation of accuracy offered by the navigation system, following patient-to-image registration (ERA), shows no correlation with true application accuracy (TRE).

The current results are similar to those from our earlier publication (2.49 ± 1.07 mm for fiducial markers and 5.03 ± 2.30 mm for surface matching) [14]. Although, since then, a number of publications have reported relatively high accuracy of surface matching systems [1, 5, 8, 9, 11]; most of these do not compare to the use of fiducial markers, and none of these report on actual measurements in a true neurosurgical setting. Instead, they use cadaver measurements, phantom measurements, calculated estimations of accuracy or measurements in non-surgical cases such as Gamma Knife treatment. Such calculations or measurements should be interpreted with caution as inaccuracy may easily be underestimated. In surgery, correct surface digitization suffers from lack of line-of-sight for the navigation system, the presence of hair and facial distortion due to loss of muscle tone, intubation, nasogastric tube placement, patient positioning, and skin movement due to use of the navigation pointer.

Part of the confusion regarding the application accuracy of neuronavigation may be due to the fact that it is not a trivial task to assess accuracy in an individual case. Some systems show a numerical value (ERA) following point-pair matching, but our current analysis demonstrates no correlation between this ERA and actual target registration accuracy, as has been demonstrated earlier for both the ERA [1, 7, 10] and root mean squared error (RMSE) [2, 4, 14]. Following surface matching, some systems show a “distance to skin surface” when the pointer is placed on the scalp. This is also of little value, as this distance only shows the error in one direction. It may be worse in another. The same is true when the surgeon runs the pointer across the scalp to check, visually, whether the navigation system indeed shows proximity to the scalp surface on the MRI images. The only sensible assessment consists of pointing at multiple targets, either adhesive markers or anatomical landmarks, and to evaluate whether the distance to these targets in the MRI images is acceptable for the type of surgery at hand. And then, still, one is not sure whether this is representative for the accuracy at deeper structures, as accuracy is not homogenous throughout the operative volume.

Nevertheless, surface matching has such strong appeal that even procedures requiring the highest possible accuracy, such as needle biopsies, are being performed with this registration technique [12], and some even regard the use of adhesive markers unacceptable [6], as it requires additional preoperative imaging. Based on our results, such claims are still not warranted.

Our study has a number of limitations. The most important one is the fact that the accuracy measured at a single adhesive marker does not represent the accuracy of the entire surgical volume. At another point in the patient’s head, the accuracy may be different; better or worse. Furthermore, the use of an adhesive marker as a target introduces inaccuracies. The image coordinates may be inaccurate because of the size of the marker, and the real-life coordinates may be inaccurate since the skin is slightly mobile. The marker is merely used because it allows a pairwise comparison between registration strategies. Finally, we omitted a comparison with point-pair matching based on anatomical landmarks. Our previous publication demonstrated this to yield accuracy comparable to that of surface matching (4.97 ± 2.29 mm) [14]. In the light of current results, this is also likely unchanged. Anatomical landmarks could, therefore, provide an adequate back-up option if adhesive markers are unavailable and surface matching fails.

Not every neurosurgical procedure requires the same level of accuracy from the neuronavigation system. Delineating the location and extent of a craniotomy for the removal of a convexity meningioma, for instance, will tolerate larger inaccuracies than a needle biopsy of a small deep-seated lesion. Thus, the level of accuracy offered by surface matching may be tolerable for one procedure but not for another. It is important that the surgeon is aware of the difference in accuracy between different patient-to-image registration accuracies and makes an informed decision to use one or the other, depending on the type of surgery at hand.

## Conclusion

The use of surface matching with current neuronavigation systems is still consistently associated with application inaccuracies that are twice as large as those offered by the use of adhesive markers. Furthermore, these inaccuracies are not reliably reflected by the accuracy estimation provided by the neuronavigation system. Although the lower accuracy offered by surface matching may be sufficient in selected cases, it is important for a neurosurgeon to make an informed choice between these patient-to-image registration strategies, depending on the type of surgery at hand.
